# Influence of tree size on the scaling relationships of lamina and petiole traits: A case study using *Camptotheca acuminata* Decne

**DOI:** 10.1002/ece3.70066

**Published:** 2024-07-19

**Authors:** Long Chen, Ke He, Peijian Shi, Meng Lian, Weihao Yao, Karl J. Niklas

**Affiliations:** ^1^ Co‐Innovation Centre for Sustainable Forestry in Southern China, Bamboo Research Institute, College of Ecology and Environment Nanjing Forestry University Nanjing China; ^2^ School of Architecture Huaqiao University Xiamen China; ^3^ School of Civil Engineering and Architecture Xiamen University of Technology Xiamen China; ^4^ School of Integrative Plant Science Cornell University Ithaca New York USA

**Keywords:** biomass allocation, diminishing returns, leaf mass per unit area, scaling relationships, tree size

## Abstract

There is a lack of research on whether tree size affects lamina and petiole biomass allocation patterns, whereas the trade‐off between leaf biomass allocated to the lamina and the petiole is of significance when considering the hydraulic and mechanical function of the leaf as a whole. Here, *Camptotheca acuminata* Decne was selected for study because of the availability of trees differing in size growing under the same conditions. A total of 600 leaves for two tree size groups and 300 leaves per group differing in height and trunk diameter were collected. The lamina fresh mass (LFM), lamina dry mass (LDM), lamina area (LA), petiole fresh mass (PFM), and petiole length (PL) of each leaf was measured, and reduced major axis regression protocols were used to determine the scaling relationships among the five functional traits. The bootstrap percentile method was used to determine if the scaling exponents of the traits differed significantly between the two tree size groups. The results indicated that (i) there was a significant difference in the LFM, LDM, PFM, PL, LMA, LFMA and PFM/LFM between large and small trees, but no significant difference in LA; (ii) the LA versus LFM, LA versus LDM, LFM versus PFM, LA versus PFM, and PL versus PFM scaling relationships of the two groups were allometric (i.e., not isometric); (iii) there were significant differences in the scaling exponents of LA versus LFM, LA versus PFM, PL versus PFM between the two groups, but there was no significant difference in the LFM versus PFM scaling relationship between the two groups of trees. The data were also consistent with the phenomenon known as “diminishing returns”. These data indicate that tree size influences leaf biomass allocation patterns in ways that can potentially influence overall plant growth, and therefore have an important bearing on life‐history strategies.

## Graphical abstract

Tree size can significantly influence the leaf traits. The overall investment costs in the mechanical and hydraulic support of leaves were greater in the larger tree size group compared to those of the smaller tree size group.

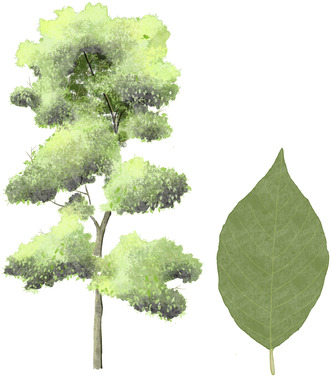

## 1 INTRODUCTION

Foliage leaves are the principal photosynthesis organs of the majority of vascular land plants (Esau, 1965), and the interplay among leaf functional traits at different life stages reflects fundamental trade‐offs in resource acquisition for growth and reproduction under specific environmental conditions (Falster et al., 2018; Jiao et al., 2024; Song & Jin, 2023). A whole leaf typically consists of an expanded lamina and a cantilevered beam‐like petiole. The leaf lamina provides a surface for light interception and carbon assimilation, whereas the petiole (along with the lamina mid‐rib in some species) serves to provide mechanical support and hydraulic continuity between the lamina and the rest of the plant body (Filartiga et al., 2022; Mencuccini, 2003; Niinemets & Kull, 1999; Niklas, 1991, 1992, 1993, 1999). In most cases, the petiole provides little toward carbon assimilation such that any biomass investment in its construction does not contribute directly to carbon assimilation (Niinemets & Kull, 1999). Similarly, excess biomass allocation to the construction of the lamina may compromise leaf stability, rendering it susceptible to environmental stresses like wind damage and drought due to inadequate support and transport through the petiole (Guo et al., 2023; Jiao et al., 2024; Li et al., 2022; Niklas, 1991; Sun et al., 2006).

Surprisingly, the importance of the leaf petiole is often neglected in comparison to that of the lamina. Yet, the petiole and the lamina are functionally interdependent in ways that profoundly influence the functionality of the entire leaf. For example, the orientation and length of the petiole can optimize leaf positioning toward the sun, thereby enhancing light‐harvesting efficiency by increasing the spatial separation of leaves and minimizing self‐shading (Niinemets, 1998; Niklas, 1991; Pearcy et al., 2005; Takenaka, 1994). However, large leaves tend to have higher transpiratory water loss due to increased surface area, and thereby require disproportionately larger investments in the vascular system and sclerenchyma within petioles to effectively transport water to mesophyll cells (Filartiga et al., 2022). Moreover, the need for stronger petioles to support greater static self‐loading and shear forces under similar wind pressure further shapes the relationship between laminas and petioles (Guo et al., 2023; Jiao et al., 2024; Li et al., 2008; Niklas, 1991, 1992). Consequently, the biomass investment between the lamina and the petiole involves many size‐dependent trade‐offs that can differ across different plant species (Li et al., 2008, 2022; Niinemets, 1998; Sun et al., 2006).

An effective and widely studied method for quantifying biomass partitioning is scaling analyses, that is, the size‐dependent relationships between two interdependent biological variables *Y*_1_ and *Y*_2_, which can be described by a power‐function taking the form $Y_{1}=\beta \;{Y_{2}}^{\alpha }$, where *β* is the normalization constant, and *α* is the scaling exponent (Milla & Reich, 2007; Niklas et al., 2007). In this framework, many of the scaling relationships among lamina functional traits (e.g., mass and area) and lamina‐petiole traits (e.g., lamina area (LA) and petiole length (PL)) have been described within and among species across altitude, leaf form, and leaf habit (Jiao et al., 2024; Li et al., 2008; Niinemets et al., 2006; Niklas et al., 2007; Thakur et al., 2018). For example, prior studies report that the scaling exponent of lamina mass versus area often exceeds unity, indicating that increases in LA fails to keep pace with the increases in lamina mass, a phenomenology called “diminishing returns” (Jiao et al., 2022; Milla & Reich, 2007; Niklas et al., 2007). One explanation for this scaling relationship is that larger leaves require more complex and effective hydraulic systems and more rigid tissues for the mechanical support of the lamina (Niinemets et al., 2007; Niklas, 2004; Runions et al., 2005). This explanation gains credibility from observations indicating that larger leaves have more massive primary and secondary vascular traces compared to smaller leaves of the same species (Guo et al., 2023; Jiao et al., 2024; Li et al., 2008, 2022). Similar albeit more complex scaling relationships between the lamina and the petiole have been observed (Levionnois et al., 2020; Li et al., 2008; Niklas, 1991, 1992; Takenaka, 1994). For example, there appears to be a “diminishing returns” in the relationship between petiole biomass and length, that is, the biomass allocated to the construction of the petiole disproportionately increases with increasing PL (Niklas, 1991). However, despite extensive research into leaf biomass allocation patterns, studies examining the scaling relationships between lamina size and petiole size are comparatively rare.

Another important but often neglected area of research is the effect (if any) of plant size on leaf biomass allocation patterns, particularly among arborescent tree species. Nevertheless, tree size (measured either by tree height or diameter at breast height, DBH) is reported to correlate with several important leaf traits and biomass allocation strategies (Liu, Hikosaka, et al., 2020; Niinemets et al., 2002; Sala et al., 1994; Tredennick et al., 2018), perhaps because the efficiency of hydraulic transport is a size‐dependent phenomenon (Mencuccini et al., 2005; Niinemets et al., 1999; Niklas, 1994, 2007; Sala et al., 1994). Larger trees tend to have larger leaf mass, leaf thickness, leaf density and leaf dry mass per unit area (LMA) than smaller trees (Burgess & Dawson, 2007; Ma et al., 2022; Niinemets, 1996; Niinemets et al., 1999; Niklas & Cobb, 2008), whereas small trees tend to have a higher net photosynthetic rate, nitrogen use efficiency, and stomatal conductance under the condition of light saturation (Song & Jin, 2023). In addition, wind speeds and thus drag forces tend to increase with increasing canopy height (Niinemets & Fleck, 2002). It is not unreasonable therefore to speculate that many of the functional traits of leaves will manifest scaling relationships that will differ even among conspecifics differing in height.

**FIGURE 1:**
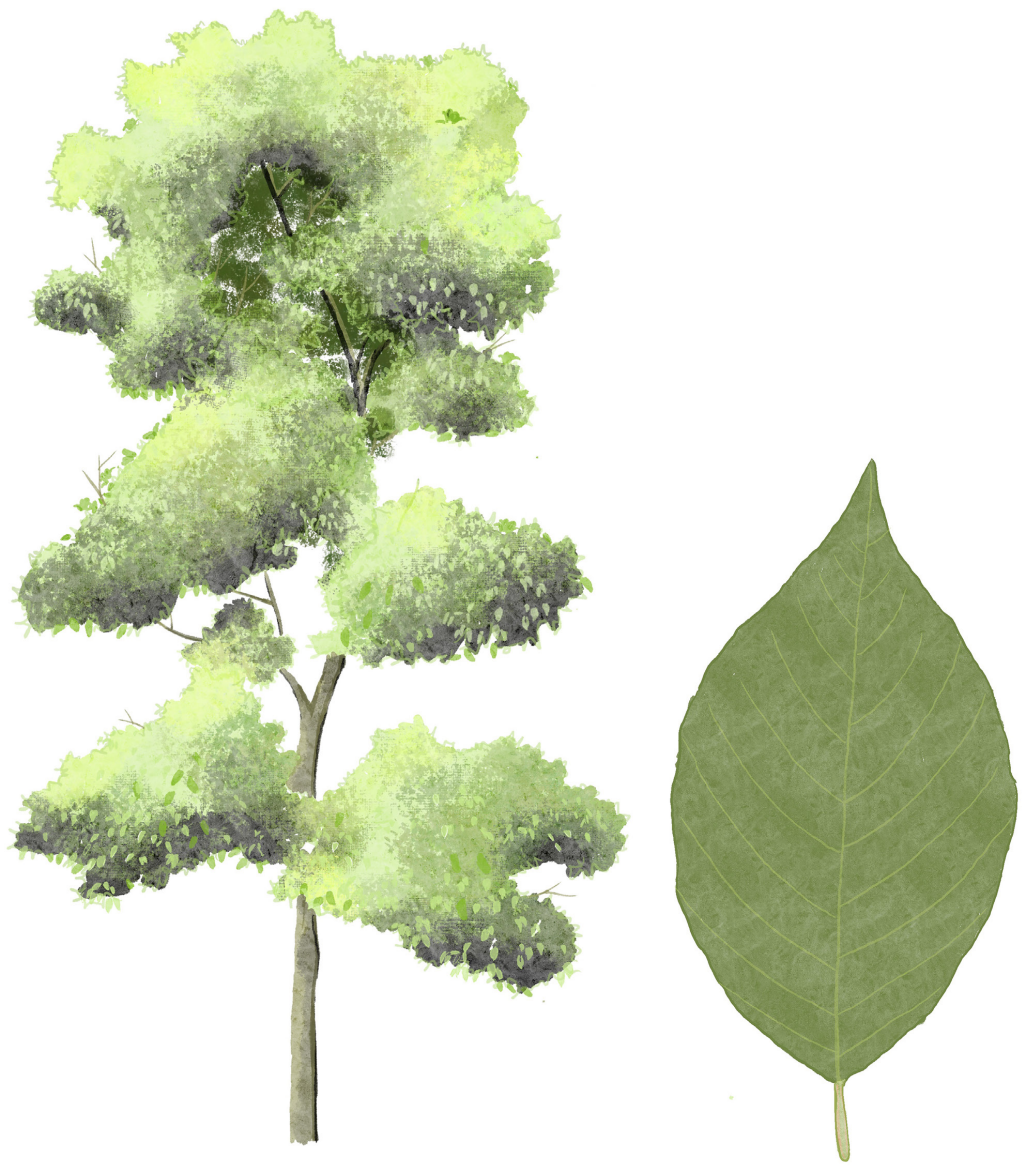
Freehand drawing of the above‐ground structure of typical *C. acuminat*e tree and a representative mature leaf.

To address this possibility, we examined a total of 600 leaves produced by two tree size groups of *C. acuminata* Decne differing significantly in tree height (300 leaves from each group). This species, which is a national second‐class protected plant in China, was selected for study because of the availability of trees differing in size but growing under nearly identical horticultural conditions and because its leaf shape is comparatively simple and thus easily quantified (Figure 1). Using 300 leaves drawn from each of the two tree size groups, we measured six functional leaf traits and sought to examine (i) whether there are robust scaling relationships among lamina and petiole traits in each of the two groups, and (ii) whether the scaling of these traits differs significantly as a function of tree size.

## 2 MATERIALS AND METHODS

### 2.1 Leaf sampling

A total of 600 mature and undamaged *C. acuminata* leaves were collected from six trees growing in the Nanjing Forestry University campus, Nanjing, Jiangsu Province, China. Table 1 provides detailed information for each of the six *C. acuminata* trees. As noted, this species was selected for study because of the availability of trees differing in size but growing under nearly identical horticultural conditions. It was also selected because its leaves are relatively simple in shape and therefore comparatively easy to measure variables such as leaf area and PL (Figure 1). In addition, the six studied trees were higher than adjacent trees from other species, so the effect of shading from other species on the leaf lamina and petiole traits of the studied trees was neglected.

**TABLE 1:** Sampling information of trees and leaves.

Tree size group	Tree code	Sampling date	Number of leaves	DBH^a^ (cm)	Height (m)	Location
Small trees	1	2023.07.18	100	15.60	11.5	118°49′17″ E, 32°04′42″ N
	2	2023.07.19	100	19.09	12.1	118°49′17″ E, 32°04′42″ N
	3	2023.07.19	100	23.25	13.7	118°49′09″ E, 32°04′38″ N
Large trees	4	2023.07.19	100	40.12	16.5	118°49′07″ E, 32°04′38″ N
	5	2023.07.19	100	42.36	18.1	118°49′07″ E, 32°04′38″ N
	6	2023.07.20	100	44.74	18.5	118°49′01″ E, 32°04′38″ N

^a^ DBH is an abbreviation of the diameter at breat hegith (cm).

A total of 300 leaves were collected from each of two groups of trees differing significantly in the tree height and diameter at the breast height (i.e., 100 leaves from each tree). Previous studies have shown that the layer of the tree canopy can affect leaf traits (Niinemets et al., 2002). Therefore, all leaves were collected from the lower canopy of each tree (see Figure 1 for a schematic of a typical *C. acuminata* canopy). Considering that the season and age of leaves may influence the mass of leaves and petioles, all of the leaves were collected in a short period of time (from July 18th to July 20th, 2023). To reduce water loss during transport, leaves were wrapped in wet paper, placed in resealable plastic bags (28 × 20 cm), and brought back to the laboratory of Nanjing Forestry University in <2 h.

### 2.2 Data acquisition

The lamina fresh mass (LFM), lamina dry mass (LDM), and petiole fresh mass (PFM) were measured using an electronic balance (ME204/02, Mettler Toledo Company, Greifensee, Switzerland; measurement accuracy 0.0001 g). The PL was measured using a ruler with a .05 cm scale accuracy. Each fresh leaf was scanned to determine leaf area (LA) using a photo scanner (V550, Epson, Batam, Indonesia). The scans were transformed into black‐white images in .bmp format by Adobe Photoshop CS6 (version 13.0; Adobe, San Jose, CA, USA). The Matlab (version ≥ 2009a; MathWorks, Natick, MA, USA) procedure developed by Shi et al. (2018) and Su et al. (2019) was then used to obtain the lamina boundary coordinates. LA was calculated using the “bilat” function of the “biogeom” package (version 1.3.5; Shi et al., 2022) based on the statistical software R (version 4.2.0; R Core Team, 2022).

### 2.3 Statistical analyses

We used the *t* test with a .05 significance level (Student, 1908) to test whether there is a significant difference in each of the leaf lamina and petiole traits (including the leaf LFM, LDM, LA, and PFM, PL, the PFM/LFM ratio, LFM per unit area, and LDM per unit area [i.e., LMA]) between the two tree size groups. There were significant differences in heights (*t* = −5.87, and *p* < .05) and diameters at breast height (*t* = −8.94, and *p* < .05) between the two groups of trees. The power function was used to describe the scaling relationships between any two variables (i.e., LA vs. LFM): $Y_{1}=\beta \;{Y_{2}}^{\alpha }$, where *Y*_1_ and *Y*_2_ represent two interdependent variables; *β* represents a normalization constant; *α* is the scaling exponent of *Y*_1_ versus *Y*_2_. To stabilize the variance of *Y*_1_, the logarithm of both sides on the power function equation of *Y*_1_ and *Y*_2_ was taken at the same time, and the following formula is usually used (Niklas, 1994; Niklas et al., 2007): y=γ+αx, where *y* = ln *Y*_1_, *x* = ln *Y*_2_ and *γ* = ln *β*. The parameters *γ* and *α* of the regression line were estimated using reduced major axis protocols (Niklas, 1994; Smith, 2009). If the interdependent variables are interchanged, the previously estimated slope is actually a reciprocal of the currently estimated slope. The bootstrap percentile method was used to test the significance of the difference in the estimated scaling exponents of *y* versus *x* between the two tree size groups (Efron & Tibshirani, 1993; Sandhu et al., 2011). All statistical analyses were performed using R (R Core Team, 2022). Reduced major axis protocols were also used to fit the combination data of the two tree size groups to test whether there were allometric relationships between LA and LFM, between LFM and PFM, between LA and PFM, and between the PL and PFM regardless of the conspecific variation in leaf traits across different tree size groups.

## 3 RESULTS

**FIGURE 2:**
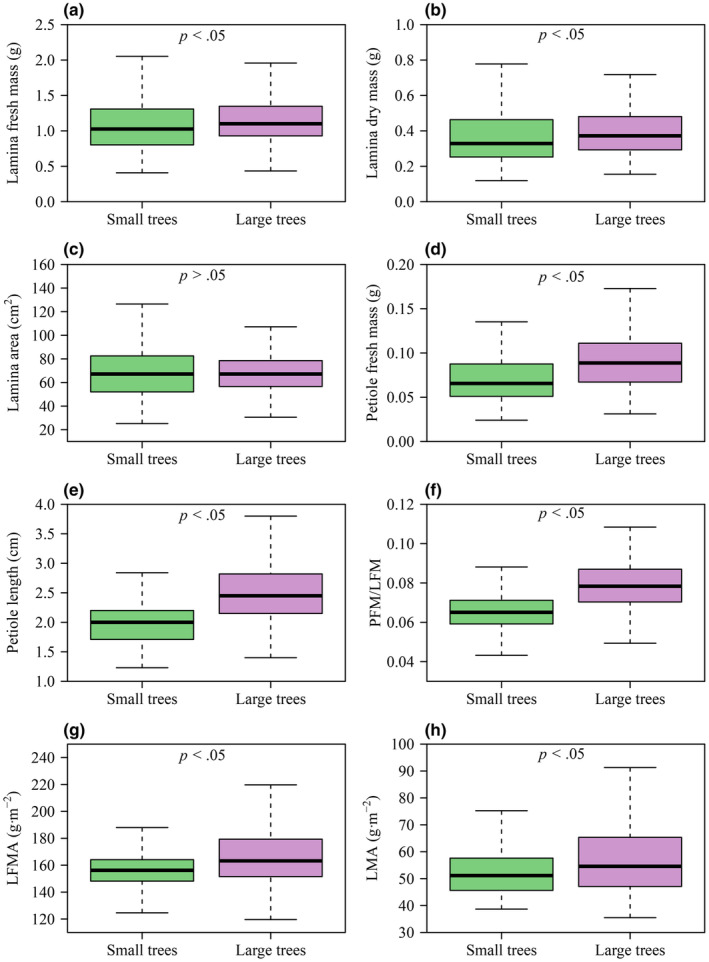
Boxplots of (a) LFM, (b) LDM, (c) LA, (d) PFM, (e) PL, (f) the ratio of PFM/LFM, (g) lamina fresh mass per unit lamina area (LFMA), (h) LMA for the two tree size groups. In each panel, there is a significant difference (*p* ≤ .05) between the two tree size groups, but no significant difference (*p* > .05) between the two tree size groups at the .05 significance level. For the label on the *x*‐axis of each panel, “Small trees” represents the smaller tree size group, and “Large trees” represents the larger tree size group.

Apart from leaf area (LA), tree size had significant effects on LFM, LDM, PFM, PL, PFM/LFM, lamina fresh mass per unit area (LFMA), and lamina dry mass per unit area (LMA) (*p* < .05). The mean values of each of the seven variables (i.e., LFM, LDM, PFM, PL, PFM/LFM, LFMA, and LMA) were significantly larger in the larger tree size group compared to those of the smaller tree size group (Figure 2), based on the *p* < .05 value of *t* tests.

**FIGURE 3:**
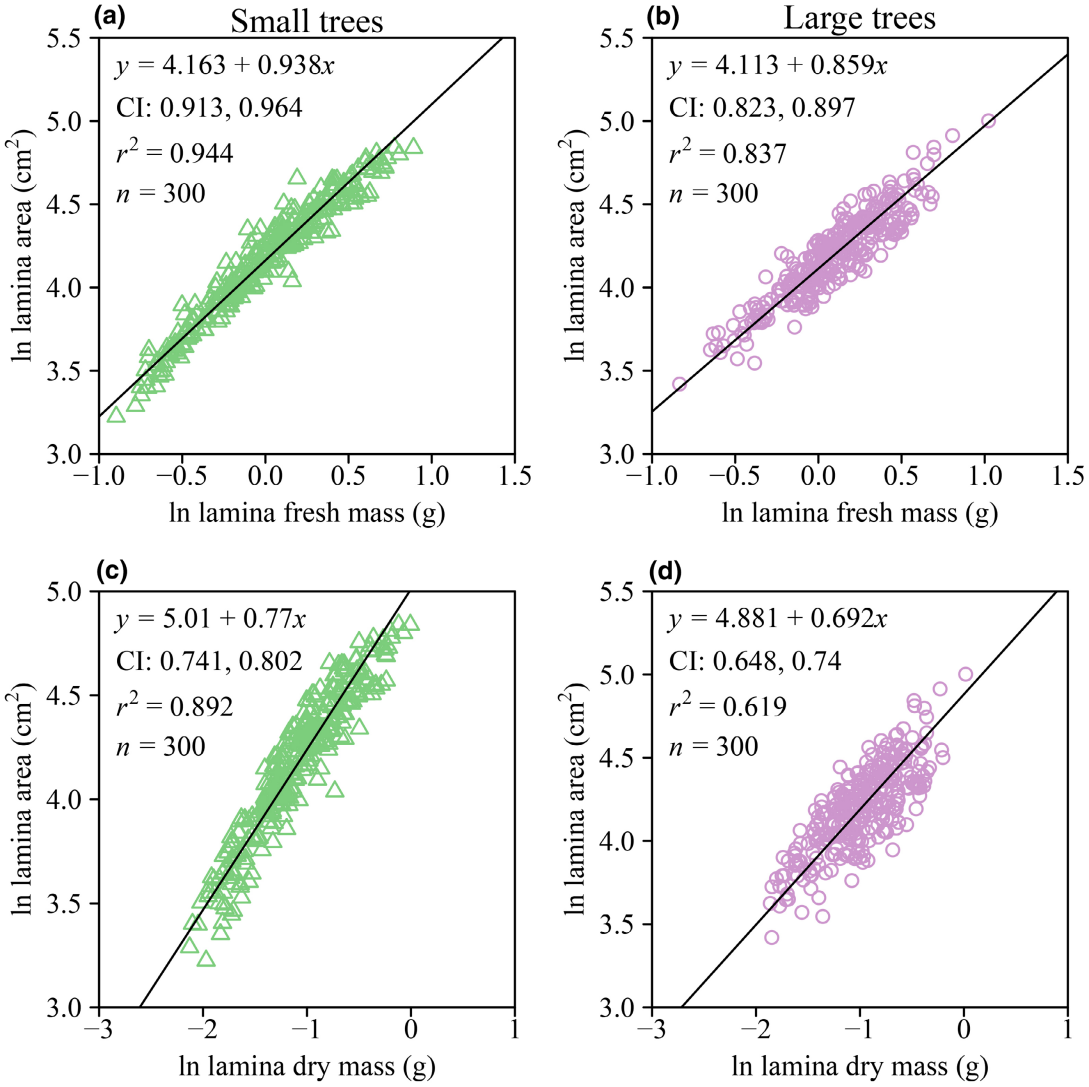
Fitted scaling relationships between the LA and LFM (a, b), and between the LA and LDM (c, d) for each of the two tree size groups. The icons represent the observations on the log–log axis. The solid lines are RMA regression lines; CI is the 95% confidence intervals of the slope (i.e., the scaling exponent); *r* ^2^ is the coefficient of determination; and *n* is the sample size.

**FIGURE 4:**
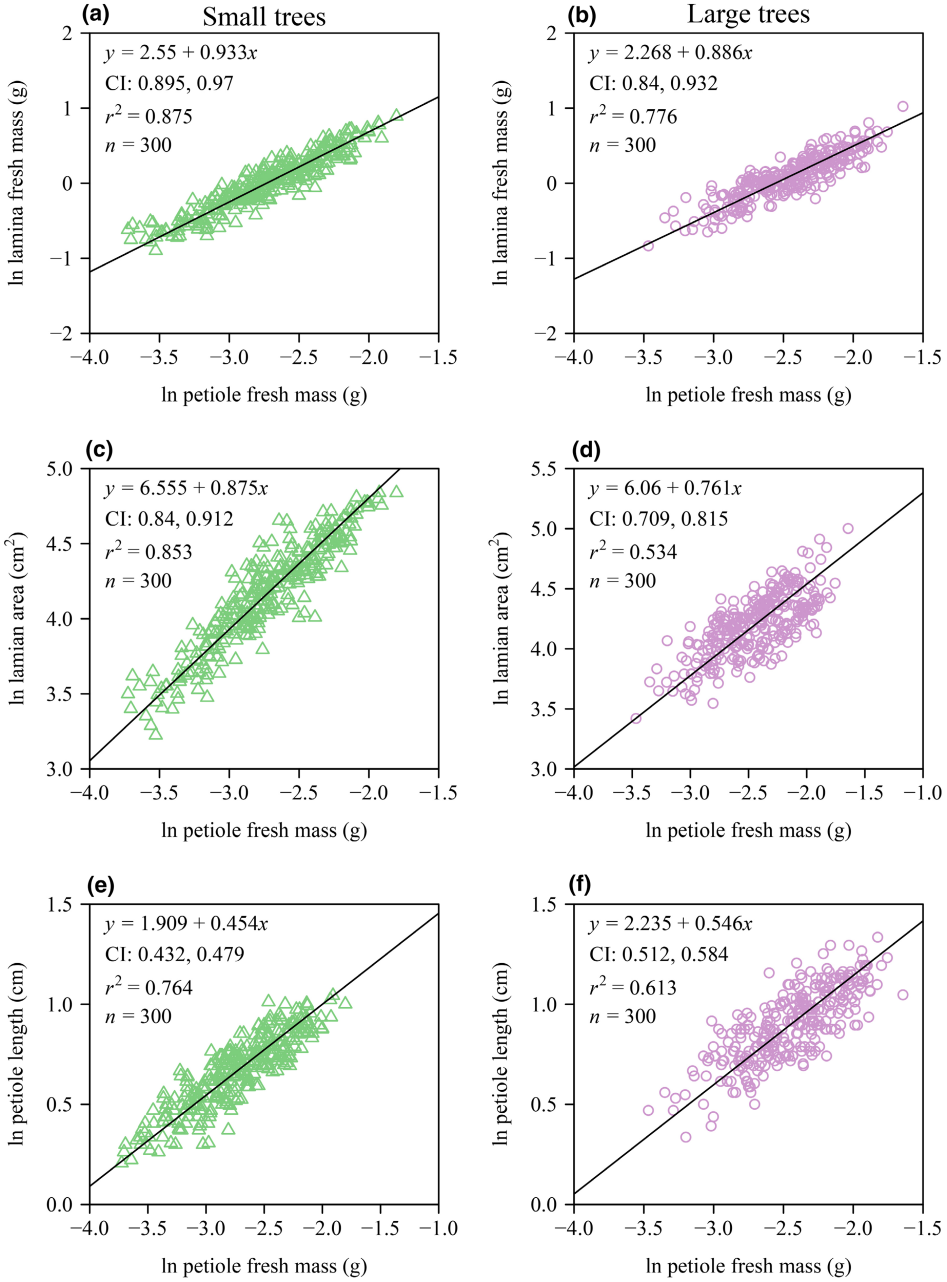
Fitted scaling relationships between LFM and PFM (a, b), between the LA and PFM (c, d), and between the PL and PFM (e, f) for each of the two tree size groups. The icons represent the observations on the log–log axis. The solid lines are RMA regression lines; CI is the 95% confidence intervals of the slope (i.e., the scaling exponent); *r* ^2^ is the coefficient of determination; and *n* is the sample size.

The numerical values of the scaling exponents of LA versus LFM, LA versus LDM, LFM versus PFM, LA versus PFM, and PL versus PFM were all smaller than unity, and the corresponding 95% CIs of each of these scaling exponents obtained by using the bootstrap percentile method did not include unity for each of the two tree size groups. Thus, increases in LA did not keep pace with increases in either LFM or LDM, increases in lamina size (as measured by both LFM and LA) did not keep pace with increases in PFM, and increases in PL did not keep pace with increases in PFM, that is, the upper bound of the 95% CI of each scaling exponent was smaller than unity (Figures 3 and 4). In addition, the scaling relationship of LA versus LFM was statistically more robust than that of LA versus LDM based on a larger coefficient of determination (Figure 3).

**FIGURE 5:**
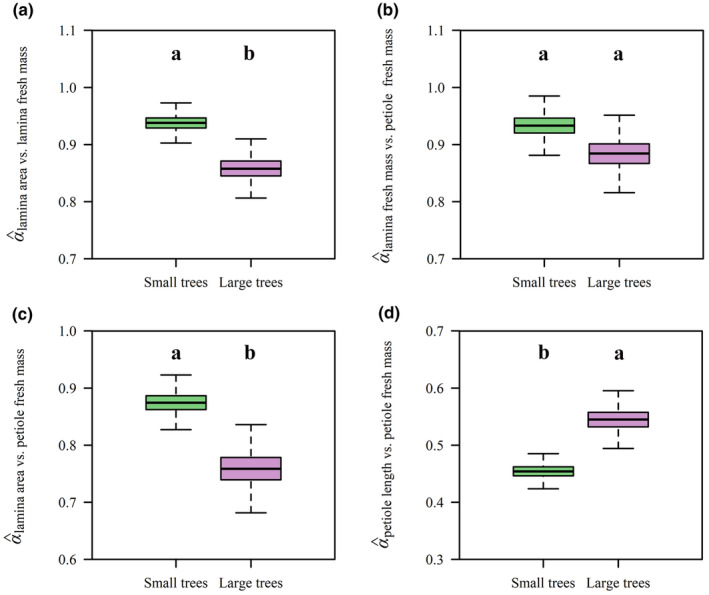
Comparisons of the scaling exponents of the LA versus LFM (a), LFM versus PFM (b), LA versus PFM (c), PL versus PFM (d) between the two tree size groups. For the label on the *x*‐axis of each panel, “Small trees” represents the smaller tree size group, and “Large trees” represents the larger tree size group. The lowercase letters indicate the significance of the difference between the two tree size groups.

**FIGURE 6:**
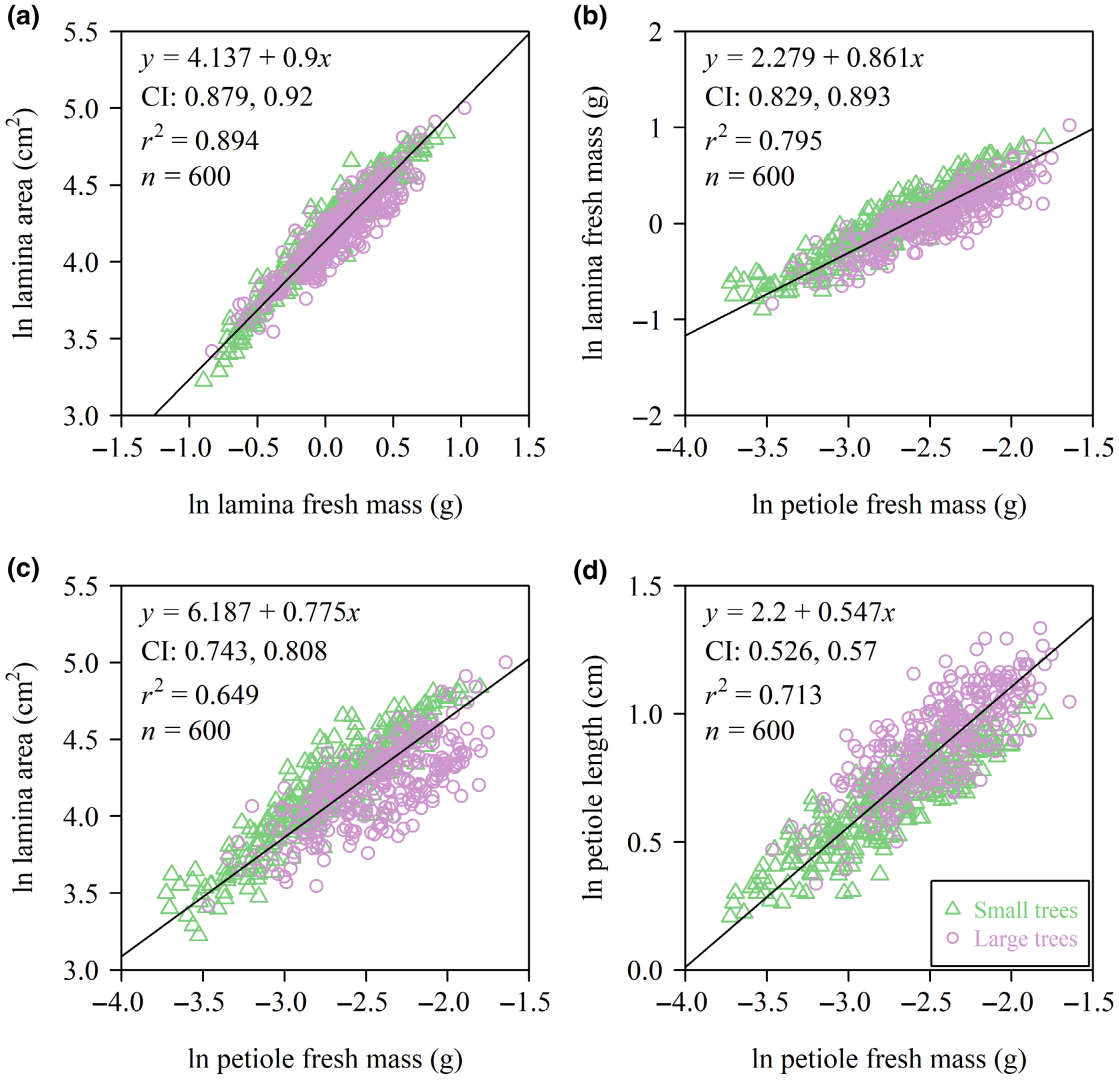
Fitted scaling relationships between LA and LFM (a), between LFM and PFM (b), between LA and PFM (c), and between the PL and PFM (d) for the combination data of the two tree size groups. The icons represent the observations on the log–log axis. The solid lines are RMA regression lines; CI is the 95% confidence intervals of the slope (i.e., the scaling exponent); *r* ^2^ is the coefficient of determination; and *n* is the sample size.

The numerical values of the scaling exponents of LA versus LFM, LA versus LDM, LA versus PFM, and PL versus PFM were statistically significantly correlated with tree size (*p* < .05) (Figure 5), with the exception of the scaling exponent of LFM versus PFM. The scaling exponents of LA versus LFM and LA versus PFM were numerically smaller in the larger tree size group compared to those in the smaller tree size group, and the PL versus PFM scaling exponent was larger in the large tree size group than that in the smaller tree size group (Figure 5). Thus, increases in LA involved larger increases in LFM and LDM among the larger tree size group compared to those among the smaller tree size group. Similarly, increases in LA required larger increases in PFM in the larger tree size group compared to those in the smaller tree size group. However, increases in PL involved larger increases in PFM among the smaller tree size group compared to those in the larger tree size group. For the combination data of the two tree size groups, the scaling exponents of LA versus LFM, LFM versus PFM, LA versus PFM, and PL versus PFM were all significantly smaller than unity given the upper bounds of their slopes’ 95% CIs were all smaller than unity (Figure 6). The results for the combination data regardless of the influence of tree size on leaf traits were in accord with those for each tree size group.

These trends were interpreted to indicate that the overall investment costs in the mechanical and hydraulic support of leaves were greater in the larger tree size group compared to those of the smaller tree size group.

## 4 DISCUSSION

The scaling relationships derived from two statistically different sized groups of trees indicate that tree size influences functionally lamina and petiole traits, that is, LFM, LDM, PFM, PL, PFM/LFM, LFMA, and LMA differ significantly between the two tree size groups. Some what surprisingly, however, there is no significant difference in LA between the two groups. Therefore, the data are not entirely in line with the hypothesis that tree size affects all of the scaling relationships between lamina size and petiole size. Taken at face value, these relationships indicate the leaf area involved in light interception is conserved regardless of tree size, but other variables of interest, such bulk tissue density and lamina thickness, are affected.

Specifically, the scaling exponents of LA versus LFM, LA versus LDM, and LA versus PFM of the larger tree size group are numerically smaller than those of the smaller tree size group, as predicted by the hypothesis. Yet, there is no significant difference in the numerical values of the scaling exponent of LFM versus PFM between the two groups. We speculate that, due to the similarity in LA, between the two tree size groups, there may be little or no difference in hydraulic demand or transport resistance. If true, the differences in the scaling relationship between LFM and PFM might be mainly determined by the necessity for mechanical support that, owing to the shading in larger canopies can be compensated by more elongated petioles. This speculation is consistent with the observations that the leaves sampled from the lower canopies of both large and small trees were significantly different in their petiolar length.

These general observations are discussed in greater detail in the following two sections.

### 4.1 Diminishing returns of LA versus lamina mass in the two tree size groups

The data from both tree size groups are consistent with the prediction of the diminishing returns hypothesis, that is, the numerical values of the scaling exponents for the LA versus LFM and LA versus LDM scaling relationships were smaller than unity (Figure 3). Therefore, regardless of the difference in the size of the two tree groups, increases in LA fail to keep pace with the increases in lamina mass, which is in agreement with previous studies (Guo et al., 2023; Jiao et al., 2024; Li et al., 2022; Niklas et al., 2007). Moreover, the numerical value of the scaling exponent of LA versus LFM in the larger tree size group is significantly numerically smaller than that of the smaller tree size group, which is an important factor in determining the degree of diminishing returns (Figure 5a), that is, it appears that the smaller trees have a lower biomass investment cost (i.e., increases in area per lamina mass investment) relative to the larger trees, which is in accord with prior reports for two other species of deciduous trees (Liu, Niklas, et al., 2020). We speculate that this is an adaptive strategy for saplings and small trees because it permits “cheaper leaves” and allows smaller trees to compete with larger conspecifics for light. Overall, the scaling relationships observed in this study are consistent with the proposition that lamina mass disproportionately increases with increasing tree size, whereas leaf area remains largely unaffected. If generally true, this result likely reflects the trade‐offs between the costs of construction and the benefits of increased leaf area across life‐forms (Niklas & Cobb, 2008). Indeed, LMA is not constant for the leaves drawn from the two tree size groups (Figure 2), indicating that either leaf‐tissue bulk density or lamina thickness increases as tree size increases (England & Attiwill, 2007; Kull & Niinemets, 1993; Niinemets, 1996).

This last speculation is consistent with a larger body of data and phenomenology. For example, the LMA of adult trees is reported to be significantly and positively correlated with tree size, and the LMA of the upper one‐third of the leaves of the canopies is reported to be greater than that of the lower one‐third of leaves (England & Attiwill, 2007; Kull & Niinemets, 1993; Niinemets, 1996). In a study of a broad‐leaved evergreen species (*Eucalyptus regnans*), England and Attiwill (2007) report that leaf area decreases and leaf thickness increases with increasing tree height. In addition, there is evidence that even in the same forest, small trees of the same species may grow larger or thinner leaves than larger conspecifics perhaps to cope with differences in canopy shading (Augspurger & Bartlett, 2003).

In turn, most studies have shown that height has a limiting effect on hydraulic resistance and that leaf water stress increases with increasing tree height (Mencuccini, 2003; Ryan et al., 2006). Thus, larger trees are predicted to be possibly more susceptible to the negative effects if drought than smaller trees (Rowland et al., 2015; Stovall et al., 2019). To cope with hydraulic limitation, it would be advantageous for larger trees to reduce leaf stomatal conductance and increase the vascular bundle redundancy in leaves to improve water transport capacity (Niinemets et al., 2007; Ryan et al., 1997). Along these lines, some studies have shown that the leaves of larger trees reduce water potential by increasing the input of solutes such as starch and lipids to obtain sufficient water, resulting in an increase in lamina mass per unit area (Sala & Hoch, 2009; Song & Jin, 2023).

In this context, it is important to recall that LMA is frequently used as a surrogate measure of photosynthetic rate and growth strategy, in part because photosynthetic capacity per unit dry mass is negatively correlated with LMA (Niinemets, 1999; Poorter et al., 2009; Wright et al., 2004). Thus, larger trees may have relatively thicker or higher‐density leaves and smaller photosynthetic returns on individual leaves compared to smaller trees (Niinemets, 1999; Wright et al., 2004). Accordingly, the scaling exponents of LA versus lamina mass are expected to change as a function of tree size as leaf development balances the requirement for hydraulic resistance and protection against embolism. Indeed, previous studies have shown that individual leaf productivity decreases with tree size and age (Stephenson et al., 2014).

### 4.2 The scaling exponents governing lamina size and petiole size in the two tree size groups

The data indicate that PFM increases disproportionately with increasing LFM and LA for each of the two tree size groups (Figure 4), which is in agreement with previous studies (Guo et al., 2023; Jiao et al., 2024; Li et al., 2008, 2022). This trend is hypothesized to reflect a mechanism to cope with the larger drag forces and static loads incurred by leaves with larger laminae (Li et al., 2022; Niklas, 1999). The data presented here show that the scaling exponent of LA versus PFM of the larger trees is significantly numerically smaller than that of the smaller trees (Figure 5c), such that larger leaves have larger and more massive petioles per LA. One explanation for this observation is that larger trees experience greater water stress as a consequence of being taller, which requires a greater hydraulic connectivity (Mencuccini, 2003; Ryan et al., 1997, 2006; Sala & Hoch, 2009; Song & Jin, 2023). However, the statistically insignificant relationship observed between PFM and LFM scaling exponents between the two groups is not in agreement with the expectation that the scaling exponents would differ between the two tree size groups (Figure 5b). However, it should be noted that the lamina mid‐rib significantly contributes to the mechanical support of the lamina (Niinemets et al., 2007; Sun et al., 2006). Previous studies have shown that the fraction of the mid‐rib within the lamina scales positively with LFM (Niinemets et al., 2007). In our study, the data indicate that the leaves of the larger trees may invest more in their mid‐rib construction, which may compensate for the investment in petiole construction, that is, the petiole and the midrib should be considered as a single mechanical “device”.

Consistent with previous studies, the relationship between PL and PFM is allometric (Figure 4) (see Niklas, 1991). Our results indicate that the relevant scaling exponents differ between the two tree size groups (Figure 5d). This observation is consistent with basic mechanical theory, which predicts that the bending moment of a cantilevered beam increases as the cube of its length provided that the bulk of the mass loading the beam is at the distal end (Gere & Timoshenko, 1997). Basic mechanical theory predicts that increasingly longer petioles require disproportionately larger biomass to support the bending moments exerted by lamina mass (Niklas, 1991, 1992, 1999; Pearcy et al., 2005). In addition, larger trees have longer petioles, which are capable of coping with stronger wind induced drag forces (Niklas, 1991, 1992; Vogel, 1989). An additional advantage is that longer petioles are capable of reducing self‐shading and optimizing light harvesting (Niinemets & Fleck, 2002; Niklas, 1988; Pearcy et al., 2005; Takenaka, 1994).

## 5 CONCLUSIONS

The data indicate that the scaling relationships for important leaf lamina and petiole traits differ as a function of tree size (as quantified by tree height or basal trunk diameter). A disproportionate biomass allocation between the leaf LA and lamina mass, and between leaf lamina size and petiole size are confirmed. Larger trees tend to have larger lamina mass, PFM, and PL, but not lamina surface area. All of the numerical values of the scaling exponents of these relationships are smaller than unity, which provides additional support for the hypothesis of diminishing returns for both tree size groups. Therefore, tree size must be included as an important variable of interest when evaluating biomass allocation patterns, which should inform future empirical studies and models for how tree functional traits affect the photosynthetic performance.

## AUTHOR CONTRIBUTIONS

**Long Chen:** Formal analysis (equal); investigation (equal); writing – original draft (equal). **Ke He:** Formal analysis (equal); writing – original draft (equal). **Peijian Shi:** Formal analysis (equal); supervision (equal); writing – review and editing (lead). **Meng Lian:** Formal analysis (equal); investigation (equal); writing – review and editing (supporting). **Weihao Yao:** Formal analysis (equal); investigation (equal); writing – review and editing (supporting). **Karl J. Niklas:** Conceptualization (lead); formal analysis (equal); supervision (equal); writing – review and editing (lead).

## FUNDING INFORMATION

The authors declare that no funds, grants, or other support were received during the preparation of this manuscript.

## CONFLICT OF INTEREST STATEMENT

The authors declare that they have no conflict of interest.

## Supporting information


**Table S1.**.

## Data Availability

The raw data of leaf lamina size (measured by the leaf LDM and fresh mass) and petiole size (measured by the PFM and length) are listed in Table S1.
